# Reactive Distillation of Glycolic Acid Using Heterogeneous Catalysts: Experimental Studies and Process Simulation

**DOI:** 10.3389/fchem.2022.909380

**Published:** 2022-06-15

**Authors:** Carole Mutschler, Juliana Aparicio, Ilham Mokbel, Mickaël Capron, Pascal Fongarland, Marcia Araque, Clémence Nikitine

**Affiliations:** ^1^ CP2M, UMR CNRS 5128, University Lyon 1, CPE Lyon, Villeurbanne, France; ^2^ University Lille, CNRS, Centrale Lille, ENSCL, University Artois, UMR 8181 - UCCS - Unité de Catalyse et Chimie du Solide, Lille, France; ^3^ LMI, UMR CNRS 5615, University Lyon 1, Villeurbanne, France

**Keywords:** vapor–liquid equilibrium, glycolic acid, butyl glycolate, reactive distillation, reaction kinetics

## Abstract

The glycerol oxidation reaction was developed leading to selective catalysts and optimum conditions for the production of carboxylic acids such as glycolic acid. However, carboxylic acids are produced in highly diluted mixtures, challenging the recovery and purification, and resulting in high production costs, polymerization, and thermal degradation of some of the products. The protection of the acid function by esterification reaction is one of the most promising alternatives through reactive distillation (RD); this technique allows simultaneously the recovery of carboxylic acids and the elimination of most part of the water. The reactive distillation, experimental and simulation, of glycolic acid was performed, based on kinetic and thermodynamic models developed. For the thermodynamic model, binary parameters of the missing couples were determined experimentally, and the non-random two-liquid (NRTL) model was selected as the most suitable to represent the binary behavior. The kinetic study of the esterification in the presence of homogeneous and heterogeneous catalysis concluded that the heterogeneous reaction can be accurately described either by a pseudo-homogeneous model or the Langmuir–Hinshelwood (L-H) adsorption model. Reactive distillation was conducted in a distillation column filled with random packing sulfonated ion-exchange resin, Nafion NR50^®^, or with extruded TiO_2_-Wo_x_. The conversion rate of glycolic acid in reactive distillation increases from 14% without catalyst to 30% and 36% using Nafion NR50^®^ and TiO_2_-Wo_x_, respectively. As opposed to the batch reactor study, the conversion rate of glycolic acid was better with TiO_2_-Wo_x_ than with sulfonated ion-exchange resin. The better performance was related to an increase in the hydrodynamics inside the column. Tests using water in the feed confirm the hypothesis by increasing the conversion rate because of the decrease in the mass transfer resistance by reducing the average diffusion coefficient. The simulation of the reactive distillation column with ProSim^®^ Plus showed that the yield of the ester increased operating at a low feed rate with reactive stripping. In the presence of water in the feed, nonreactive stages are required, including an enrichment region to separate water vapor.

## 1 Introduction

Glycerol is generally used as an additive or as a raw material in a wide variety of processes, including the production of food additives, tobacco, and pharmaceuticals, and synthesis of trinitroglycerin, alkyd resins, and polyurethanes. It is also used in the manufacture of lacquers, varnishes, inks, adhesives, synthetic plastics, regenerated cellulose, explosives, and other industrial uses (Veluturla et al., 2018). Despite its versatility, new ways of using glycerol have been proposed. Among these valorizations, the oxidation of glycerol in aqueous solution is an interesting alternative, leading to a mixture of not only carboxylic acids including oxalic and formic acids, but also α-hydroxy acids such as lactic, glycolic, glyceric, and tartronic acids (Skrzyńska et al., 2014). Using classical noble metals such as Au and Pt, the main products are glyceric and tartronic acids. The selectivity of the reaction turns to glycolic acid when Ag-based catalysts are used (Skrzyńska et al., 2016). In recent years, the applications (hence the economic potential) of glycolic acid have increased. The molecule has two functionalities: an alcohol group and a moderately strong acid group. These qualities make the glycolic acid perfect for a wide range of applications, for example, in the pharmaceutical industry, skin care products, and the food industry—as a flavoring agent and preservative—in adhesives and plastics, and in the textile industry as dyeing and in organic synthesis (Bianchi et al., 2005; Skrzyńska et al., 2016). However, the product obtained after the catalytic reaction is a highly diluted mixture of at least three acids: glyceric, glycolic, and formic acids. In the case of Ag-based catalyst, 69% of selectivity toward glycolic acid were reached up, with the formation of 20% of formic acid and 10% of glyceric acid. An increase in the initial glycerol concentration from 0.3 to 1.5 M, a full transformation of glycerol, was achieved with the production of 42% of glycolic acid, glyceric acid, and formic acid (15%). CO_2_ production was also reported (Tavera Ruiz et al., 2021).

The production of these acids from renewable resources is of growing interest; however, new issues regarding separation emerge with the use of these resources. Unlike the petrochemical industry, the mixtures involved are highly diluted. The efficient elimination of water is a major issue in this type of process. In addition, the nonselectivity of the reactions and impurities present in the raw materials lead to the formation of coproducts and the production of highly complex mixtures. The separation processes from the petrochemical sector, such as distillation and extraction, are difficult to use and expensive. The separation by extraction or distillation is limited by the phase separation and the distribution of the components involved in the system. Therefore, the costs associated with the recovery, concentration, and purification of these carboxylic acids can represent 60%–70% of the cost of the product, making these technologies unviable.

In order to provide an integrated process, the use of reactive distillation (RD) is envisaged. It combines both reaction and separation within the same equipment. This process is clearly part of the current process evolution toward the design of multifunctional, compact, and performance-enhancing devices. RD is applied specifically to reversible chemical reactions in the liquid phase, in which reaction equilibrium limits the conversion rate of the reactants (Talnikar and Mahajan, 2014). Reactive distillation (RD) has been proposed as a promising technique for the recovery of a short-chain carboxylic acid with high purity and high yield (Kumar et al., 2006). RD improves selectivity, increases conversion rate, allows a better heat control and an effective utilization of reaction heat, increases the scope for difficult separations, and helps to void azeotropes. As the products in RD are continuously separated from the reaction zone, no limiting chemical equilibrium can be established, and thus, the reaction is maintained at a high rate, resulting in higher yields. Other benefits of RD can include the minimization of side reactions and the utilization of the reaction heat for the mass transfer within the same column. Therefore, by acting simultaneously on the distillation and reaction (RD), both investment and operating costs can be reduced compared with conventional processes and can yield benefits such as reduction in recycling, separation optimization, and lower requirements of pumps, instrumentation, and piping (Talnikar and Mahajan, 2014) (Komesu et al., 2015).

The design of the process is based on the knowledge and understanding of the chemical reaction, the phase equilibrium, and the feasibility analysis of reactive distillation. Then, the reactive distillation column is designed and synthesized to specify the configuration of the column (number of theoretical stages, position of the reactive zone, number and position of the feed, etc.) and the operating parameters of the process (reflux rate, heating power, etc.).

Esterification reactions have been repeatedly carried out in reactive distillation processes. However, the esterification of alpha-hydroxy acids in catalytic distillation has been little investigated except for the recovery of lactic acid from fermentation by esterification (Rao et al., 2014; Kumar and Mahajani, 2007). Reactive distillation (RD) has also been used for the recovery of lactic acid from aqueous solution, using different alcohols for the esterification (e.g., ethanol, butanol, methanol, and 2-propanol) (Komesu et al., 2017; Liu et al., 2006). This work presents the experimental and simulation study of the reactive distillation of glycolic acid by esterification with butanol (Rxn. 1).

$$C_{2}H_{4}O_{3}+C_{4}H_{10}O↔C_{6}H_{12}O_{3}+H_{2}O$$ (Rxn1)

Both kinetic and thermodynamic models were developed. The reaction kinetics were determined using heterogeneous catalysts, and the catalytic results were compared with those obtained using homogeneous sulfuric acid (H_2_SO_4_). The thermodynamic model was fitted using vapor–liquid and liquid–liquid equilibrium data, and the coefficients were also used for the estimation of the kinetic model *via* the fitting of the activities. This study also presents experimental results obtained on a continuous distillation pilot, where the influence of the flow rate, feed composition, reflux rate, and the catalyst mass was studied. Finally, the experimental results were compared with the simulation results of equilibrium stagewise model using ProSim^∗^ Plus, and an optimization of operating parameters was proposed.

This work develops—from bench to pilot scale—the efficient implementation of a separation technology in a more cost-effective and environmental-friendly manner, which reduces the water content before the implementation of more complex technologies for the separation of a highly diluted mixture of carboxylic acids. The implementation of separation and reaction in a single unit has high potentials, and the development of this type of technologies will accelerate the shift between petrochemical reagents and the biomass-producing pathways.

## 2 Materials and Methods

The design and simulation of the reactive distillation column for the production of butyl glycolate was performed based on four different studies: 1) thermodynamic equilibria, in order to determine the activity coefficients used for the separation and reaction calculations; 2) K_Eq_. and kinetic study, in order to determine the feasibility of reactive distillation and to integrate these studies in simulation; 3) reactive distillation experiments with a laboratory column; and 4) column simulation, in order to investigate the use of a conventional equilibrium stage model to simulate reactive distillation columns and determine the best configuration to optimize the RD process with this chemical system. The materials used for the experimental part in the first three studies are summarized in Supplementary Table S1.

### 2.1 Thermodynamic Measurements

The esterification reaction of glycolic acid with butanol (Rxn. 1) represents six different binaries: 1-butanol–butyl glycolate (binary 1); 1-butanol–water (binary 2); butyl glycolate–water (binary 3); butyl glycolate–glycolic acid (GA) (binary 4); 1-butanol–glycolic acid (binary 5); and water–glycolic acid (binary 6). Binary interaction parameters are only reported for binary 2, and the other equilibriums have not yet been assessed in the open literature. Besides, few experimental data concerning the saturated vapor pressure of butyl glycolate are available. Thus, vapor pressure of BG was determined and vapor–liquid equilibrium (VLE) was proposed for the determination of the binary interaction parameter of binary 1. Liquid–liquid equilibrium (LLE) was used for the determination of interaction parameters of binary 3, and solid–liquid equilibrium (SLE) was used for the determination of the binary interaction parameters of binaries 4, 5, and 6. For this study, the purity of the chemicals was verified by gas chromatography and a vacuum distillation was performed for the butyl glycolate (BG) in order to increase the purity to 99 wt.%.

#### 2.1.1 Vapor Pressure

**FIGURE 1:**
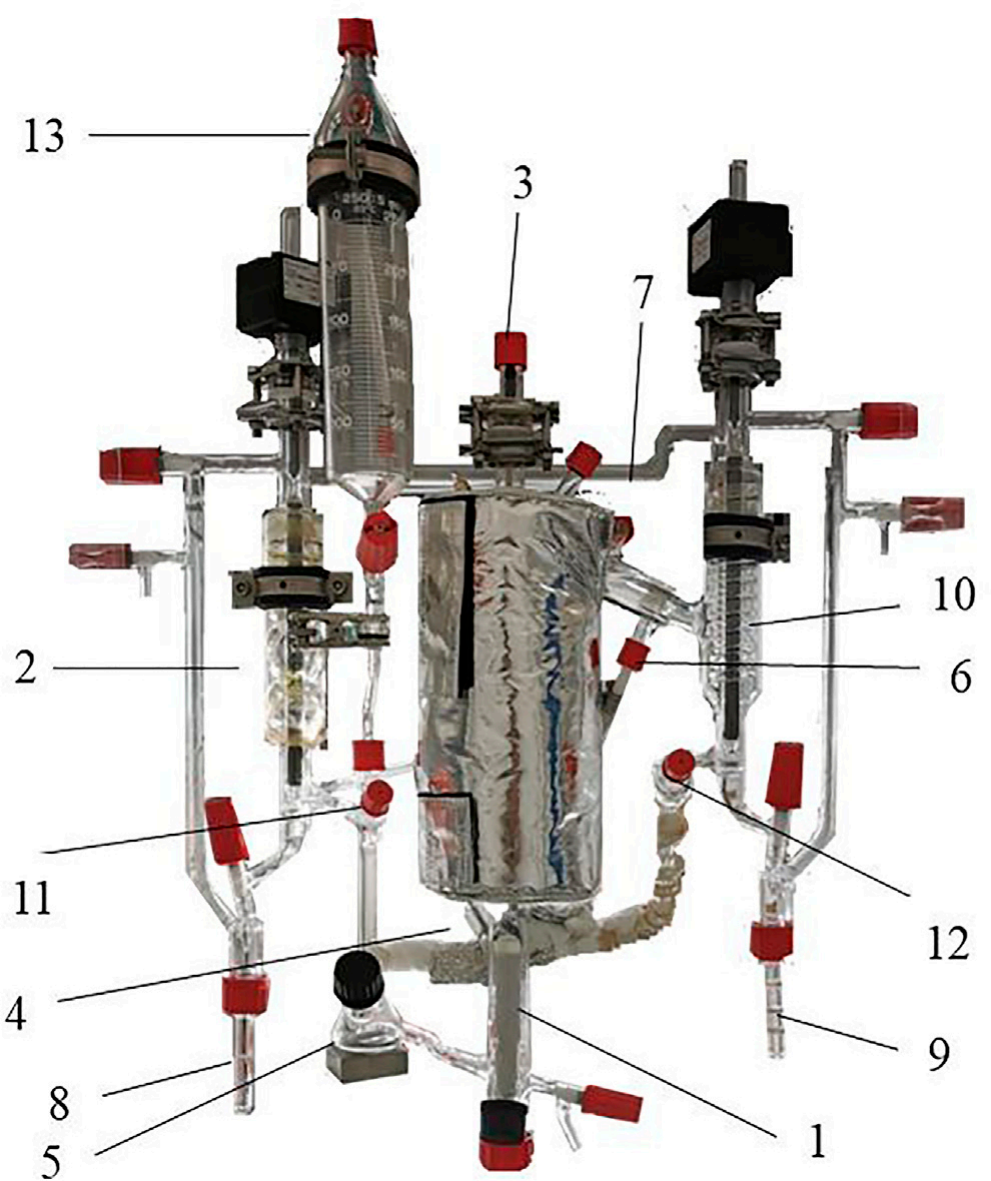
Apparatus FISCHER^®^ LABODEST^®^ VLE 602 used in this work. 1: Reservoir with immersed rod heater. 2: Condenser. 3: Vapor-phase temperature sensor. 4: Liquid-phase temperature sensor. 5: Mixer chamber. 6: Coolant water connection. 7: Pressure control line. 8: Liquid sampling port. 9: Vapor sampling port. 10: Condenser. 11: Liquid-phase septum. 12: Vapor-phase septum. 13: Side reservoir.

The vapor pressure of BG was measured in two different equipment: From 1 to 79 mbar, a static device was used and described in another publication (Mokbel et al., 1995); and from 100 to 1013.25 mbar, the measurements were performed in the FISCHER^∗^ LABODEST^∗^ VLE 602 unit (Figure 1).

The VLE 602 unit is equipped with a COTTRELL pump, and it is based on the principle of the circulation method, assuring the contact between phases and a quickly reach of the equilibrium. For the vapor pressure measurements, the mixed chamber (5, Figure 1) and the side reservoir (13, Figure 1) were filled with 80–90 ml of the pure component. The determination of the equilibrium points was performed by fixing the pressure to 10 kPa and increasing the heating power in order to obtain one or two drops per second. The equilibrium conditions were considered to be attained when the variation in temperature was lower than 0.1 K during 30 min. Once the equilibrium condition was attainted, a sample of liquid was taken (40 µl) for HPLC analysis. Before the test, the unit was degassed and dried under vacuum (25 mbar) during 1 h. Then, a flow of nitrogen was introduced and the starting working pressure was set.

#### 2.1.2 Vapor–Liquid Equilibrium

For BuOH-BG binary, VLE measurements at three pressures were carried out: 300.00, 700.00, and 1013.25 mbar. The measurements were performed in the FISCHER^®^ LABODEST^®^ VLE 602 unit as previously introduced. The mixed chamber (5, Figure 1) and the side reservoir (13, Figure 1) were filled (80–90 ml) with the less volatile component, and the second pure component is added *via* liquid-phase septum (11, Figure 1), according to previously fixed quantities. Before test, the unit was degassed and dried under vacuum (25 mbar) during 1 h. Then, a flow of nitrogen was introduced, the starting working pressure was fixed, and the heating and stirring systems were turned on. Once the equilibrium condition was attainted, samples of liquid and condensed vapor were taken (40 µl) for HPLC analysis. The accuracy of the temperature and the pressure measurements were ± 0.01 K and ± 0.1 mbar, respectively, as indicated by the supplier.

#### 2.1.3 Liquid–Liquid Equilibrium

LLE data for BG-W binary were determined using magnetically agitated vials of 30 ml, placed in an isothermal oil bath equipped with a temperature control system. Mixtures of different compositions were prepared and stirred at a constant temperature for 1 h. Then, the temperature of the mixture was measured, using an electronic thermocouple (± 0.1°C), and the samples were centrifuged for 2 min at 4000 rpm. The two liquid phases were recovered separately and weighed. The composition of the samples was measured by HPLC. Samples of 400 µl were taken and diluted in 10-ml volumetric balloons with a solution of 42%–58% volume W-CAN.

#### 2.1.4 Solid–Liquid Equilibrium

For solid–liquid equilibrium data, solubility measurements were performed. The solubility was measured by preparing a GA/solvent saturated mixtures in a 30-ml glass vial. The mixture was then immersed in an oil bath equipped with a temperature control system and agitated for 1 h. After agitation, the temperature was measured inside the vial, with an electronic thermocouple (± 0.1°C), and the liquid phase was recovered and centrifuged at 4000 rpm for 2 min. A sample of 400 µl was taken and weighed for HPLC analysis.

### 2.2 Batch Measurements: K_Eq_. and Kinetic

In general, GA esterification was carried out in a 250-ml three-necked flask, open to the atmosphere and equipped with a cooling system. The flask was placed in an oil bath under magnetic stirring, with a temperature control of within ±0.5°C. First, GA was solubilized in n-butanol at 50°C and the time zero of the reaction “t_0_” was considered when the catalyst was added, just before the reaction temperature was reached (T = T_reaction_−2°C). For K_Eq_. tests, the reaction was performed during 20 h using H_2_SO_4_ (0.257 g). The molar ratio of butanol to glycolic acid was 3, and the reaction was performed at three temperatures: 50°C, 60°C, and 70 °C. For the effect of the catalyst, the esterification was carried out at 70°C with a molar ratio of butanol to glycolic acid of 3. The reaction was performed during 4 h under reflux at constant temperature and stirring. The samples were taken at 10, 20, 30, and 60 min after t_0_ and then every hour for 4 h. H_2_SO_4_ was used as a homogeneous catalyst (0.257 g), and cation-exchange acid resins (Amberlyst 15, Amberlyst 16, Amberlyst 36, Nafion, and Dowex) were used for the heterogeneous catalytic tests (1.32 wt.%). The kinetic study was performed at different conditions: 50°C–70°C; molar ratio of butanol to GA of 1:1; 1:3; 1:6, and 1:10; and catalyst quantity of 0.3–1.5 wt.%.

For diffusional studies, the stirring rate ranged from 300 to 700 rpm, keeping the other operating parameters constant. The reaction was performed during 4 h under reflux at 70°C with Amberlyst 36 and Nafion NR50 and a molar ratio of butanol to GA of 1:10. The effect of intra-particle diffusion in the reaction was studied for the Amberlyst 36. Two different particle sizes were screened, between 250 and 500 μm and greater than 500 μm. The same reaction conditions as the one previously mentioned were used for both tests.

### 2.3 Reactive Distillation

**FIGURE 2:**
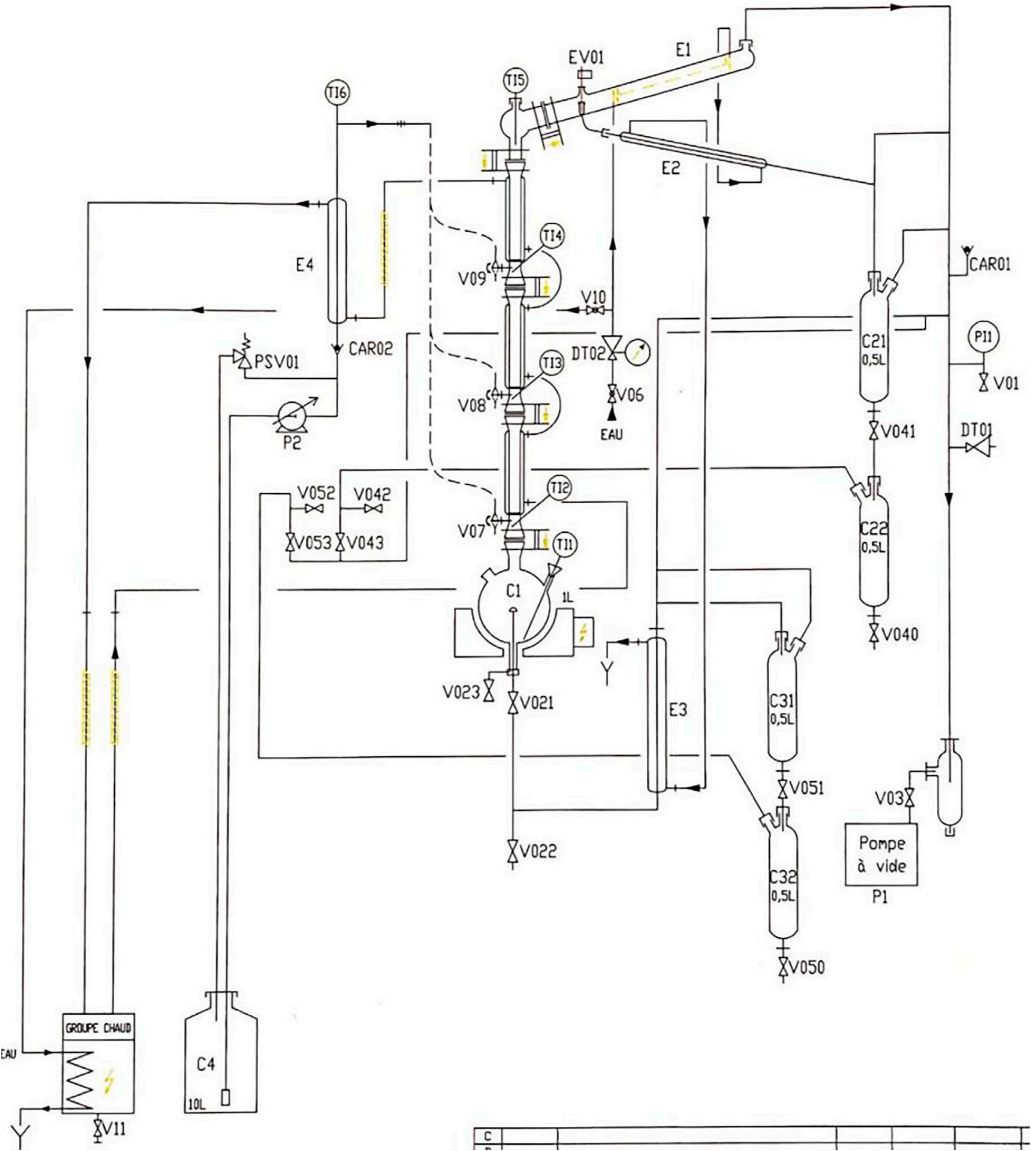
Reactive distillation pilot design by PIGNAT^®^.

The experimental setup for reactive distillation studies was performed in a pilot-scale column illustrated in Figure 2. The column consists of three glass sections with an internal diameter of 32 mm and a packing height of up to 25 cm for each segment. The setup includes a total condenser, an electronic reflux splitter for reflux ratio control, and a reboiler (1 L) with an overflow outlet and a maximum heating capacity of 450 W. A heat carrier fluid (ethylene glycol) flowing in an outer wall aims to reduce heat losses in the length of the column. Several ports in the entire column, from boiler to condenser, allow internal temperature measurement. Additionally, the assembly has a pump allowing to feed the column in stages 2 and 3 after passing through a preheating system. All experiments were performed in reduced pressure at 370 mbar, and other conditions are indicated in Table 1.

**TABLE 1:** Operating conditions for reactive distillation and results obtained with m_AG_/m_BuOH_ 1/10 g/g.

Feed					Boiler					
F (kg h^−1^)	Mass fraction			R	m_cata_ (g)	Flow (kg.h^−1^)	Mass fraction			X_exp_ (%)
	GA	Water	BuOH				GA	BG	BuOH	
0.6	0.1	0	0.9	1	8	0.549	0.081	0.046	0.873	22
0.6	0.1	0	0.9	1	8	0.552	0.079	0.044	0.877	23
0.6	0.1	0	0.9	1	17	0.451	0.075	0.057	0.868	29
0.5	0.1	0	0.9	0.5	17	0.416	0.076	0.063	0.861	30
0.5	0.1	0	0.9	0	17	0.395	0.078	0.077	0.845	34
0.5	0.1	0	0.9	5	17	0.499	0.069	0.056	0.875	28
0.6	0.1	0	0.9	1	35	0.486	0.054	0.086	0.86	43
0.6	0.1	0	0.9	1	22	0.522	0.063	0.070	0.867	30
0.2	0.1	0	0.9	1	22	0.212	0.053	0.075	0.872	42
0.2	0.084	0.044	0.872	1	22	0.158	0.056	0.1	0.844	47
0.2	0.084	0.100	0.816	1	22	0.099	0.074	0.159	0.767	54
0.2	0.073	0.147	0.780	1	22	0.07	0.080	0.217	0.703	65

### 2.4 Analysis

The samples obtained in the kinetic, VLE, LLE, and solubility measurements were analyzed by a high-performance liquid chromatograph (HPLC, SHIMADZU) with a refractive index detector (RID). The HPLC was equipped with a column Luna Omega C18 (octadecyl, inverse phase), 250 mm in length, 4.6 mm as an internal diameter, and a particle size of 5 µm. The mobile phase was acetonitrile/water 58–42 (%v/v) and acidified 0.06 g L^−1^ with respect to water, at a constant flow rate of 0.5 ml.min^−1^. The column oven temperature was kept constant at 30°C for 15 min. The volume injected for each analysis was 10 μl. The samples were diluted in a solution of equal concentration of the mobile phase, without acidification. The calibration of the products was carried out in triplicates to obtain the repeatability within 0.5% in moles. Acetonitrile (HPLC grade, EMD) was used in the HPLC analysis.

The concentrations obtained by HPLC analysis allowed for calculating the evolution of the molar concentration. From the data obtained, conversion (X) and yield (Y) were calculated by the following equations:

$$X\;=\frac{n_{GA}^{ini}-n_{GA}}{n_{GA}^{ini}}$$ (1)

$$Y=\frac{n_{BG}}{n_{GA}^{ini}}$$ (2)

where $n_{GA}^{ini}$ and $n_{GA}$ represent the moles of acid at t = 0 and at the corresponding sampling time; $n_{BG}$ corresponds to moles of butyl glycolate produced. Due to the lack of an instrument capable to determine the water amount such as Karl Fischer, the mass balance could not be determined. However, to corroborate the reliability of the analysis method, a carbon balance was completed as illustrated by Eq. 3.

$$CB\left(\%\right)=\frac{\left(2n_{GA}^{ini}+4n_{BuOH}^{ini}\right)-\left(2n_{GA}+4n_{BuOH}+6n_{BG}\right)}{\left(2n_{GA}^{ini}+4n_{BuOH}^{ini}\right)}$$ (3)

Determination of acidic sites in water for two resins was carried out using an acid–base feedback method described by Vilcocq et al. (2015). With an acid site number of 0.88 mol/g, the Nafion NR50^∗^ ion-exchange resin has a catalytic potential of interest for the reaction studied.

### 2.5 Column Simulation

Two simulations studies have been performed. The first one is a feasibility study based on reactive residue curves. The reactive residue curve map (rCRM) is a very useful tool to obtain conceptual designs of reactive distillation columns. The methodology for determining them has already been described (Niang and Mikitenko, 1998; Barbosa and Doherty, 1988). Such maps allow us to verify the existence of stable nodes and to predict the numbers of feed and their positions. The kinetic model is incorporated into the differential equations for the computation of the reactive residue curve as:

$$\frac{dX_{i}}{dt}=\frac{L}{V}\left(X_{i}-Y_{i}\right)$$ (4)

where X_i_ and Y_i_ are the transformed molar fraction in the liquid and vapor phase, respectively.

For this reactive system, BG was chosen as a reference compound. Thus, the three other transformed fractions (GA, n-butanol, and water) are calculated as below:

$$X_{i}=x_{i}-\nu_{i}x_{ref}$$ (5)

$$Y_{i}=y_{i}-\nu_{i}y_{ref}$$ (6)

The pressure is fixed at 380 mbar, and thus, the degree of freedom of the system is equal to 2. Therefore, by choosing two independent variables X_BuOH_ and X_GA_, it is possible to solve the differential equations (Nc-1) for k initial coordinate points using the MATLAB software interfaced with the Simulis ProSim software for liquid–vapor equilibrium resolution.

The second one is a simulation of the RD process. To represent the continuous reactive distillation system, a model was developed with the ProSim^®^ Plus software. The thermodynamic data obtained in this work were implemented in the NRTL model of the software. An equilibrium stage model was considered, and the developed kinetic law was implemented *via* a cape open source file. It is worth mentioning that nonequilibrium models normally provide more details and more precise information to the simulation than the equilibrium models in the case of conventional packed distillation columns. However, the availability of reliable mass transfer correlations for the catalytic packing would be a prerequisite for the use of a nonequilibrium stage model. Such models and correlations are not available in the software used. In addition, the diffusional limitations of L/S mass transfer are not taken into account in the software. To overcome this problem, it was required to reduce the amount of catalyst in the reaction stage, relative to the amount of catalyst used in the experiments. Finally, in order to validate the simulation parameters, the number of equilibrium stages was set to 4 as obtained experimentally, and the conversion rate, the ester recovery rate, and this purity were compared for the same feed, distillate, and residue flow rates.

## 3 Results

### 3.1 Thermodynamic Measurements

#### 3.1.1 Vapor Pressure of Butyl Glycolate

The experimental and so far reported values for the BG vapor pressure are summarized in Supplementary Table S2. A regression analysis of Antoine equation parameters (Eq. 7) was performed by minimization of the relative least sum of squares defined in Eq. 8.

$$lnP{}^{\circ}\left(T\right)=A-\frac{B}{T+C}\;$$ (7)

$$\sigma =\;\sum {\left(\frac{P_{exp}-P_{cal}}{P_{exp}}\right)}^{2}$$ (8)

**FIGURE 3:**
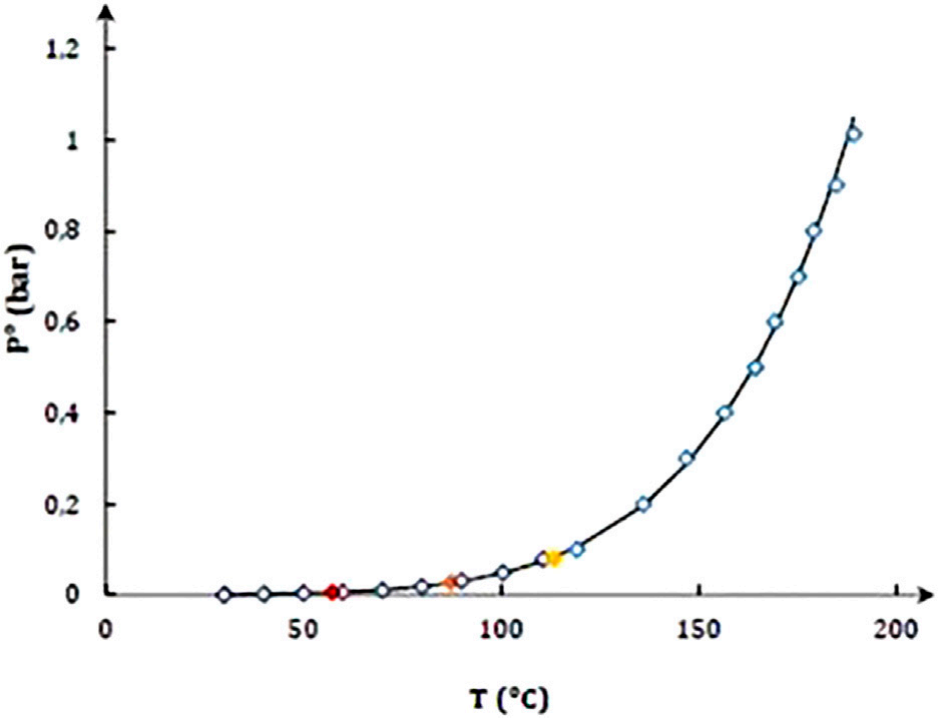
BG vapor pressure reported by Crosby and Berthold (1960) (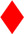), reported by Forman et al. (1941) (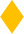), measured in FISCHER^®^ LABODEST^®^ VLE 602 (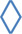), measured in LMI (◊), and calculated (---).

In Eq. 7, *P°(T)* represents the vapor pressure reported in bar, *T* is the corresponding equilibrium temperature in Kelvin, and *A*, *B*, and *C* are the Antoine equation parameters. The parameters regressed are 11.94 ± 0.3, 5140 ± 247, and −30.52 ± 8, respectively. The standard deviation associated is 0.837. The predicted and measured/reported vapor pressure values were compared (Figure 3).

As observed, it is a good agreement between the reported and the measured data, and between the measured data and the calculated values using the regressed parameters. The maximum deviation between the measured and calculated vapor pressure was of 26 Pa between 30°C and 190°C.

#### 3.1.2 Vapor–Liquid Equilibrium of Butanol–Butyl Glycolate System

The activity coefficients $\left(\gamma_{i}\right)$ of the mixture BuOH_BG were determined according to the simplified equilibrium equation at moderate pressures (Eq. 9).

$$\gamma_{i}=\frac{\varphi_{i}y_{i}P}{x_{i}P^{{}^{\circ}}\left(T\right)}$$ (9)

P is the total pressure of the system, $x_{i}$ and $y_{i}$ are the liquid and vapor molar fractions, respectively, and $\varphi_{i}$ is the fugacity coefficient of component i in the mixture.

Ideal behavior was assumed for the gas phase, given the low working pressures, and the fugacity coefficient was set to 1. The VLE measured data and the calculated $\gamma_{i}$ are summarized in Supplementary Table S3. In all cases, the consistency of the VLE data was verified using the tool for thermodynamic consistency available in Aspen Plus V10. For the three pressures, the consistency test—similar to the Redlich–Kister total area test (Duran et al., 2013)—was passed with tolerance intervals lower than 10%.

The VLE data were correlated by the NRTL thermodynamic model (Renon and Prausnitz, 1968) using the Aspen Plus V10 regression tool. The interaction parameters were adjusted by minimizing the maximum-likelihood objective function (Eq. 10), where n and σ are the number of data points and the standard deviation, respectively.

$$OF=\sum_{i=1}^{n}\left[{\left(\frac{T_{i}^{exp}-T_{i}^{cal}}{\sigma^{T}}\right)}^{2}+{\left(\frac{x_{i}^{exp}-x_{i}^{cal}}{\sigma^{x}}\right)}^{2}+{\left(\frac{y_{i}^{exp}-y_{i}^{cal}}{\sigma^{y}}\right)}^{2}\right]$$ (10)

**FIGURE 4:**
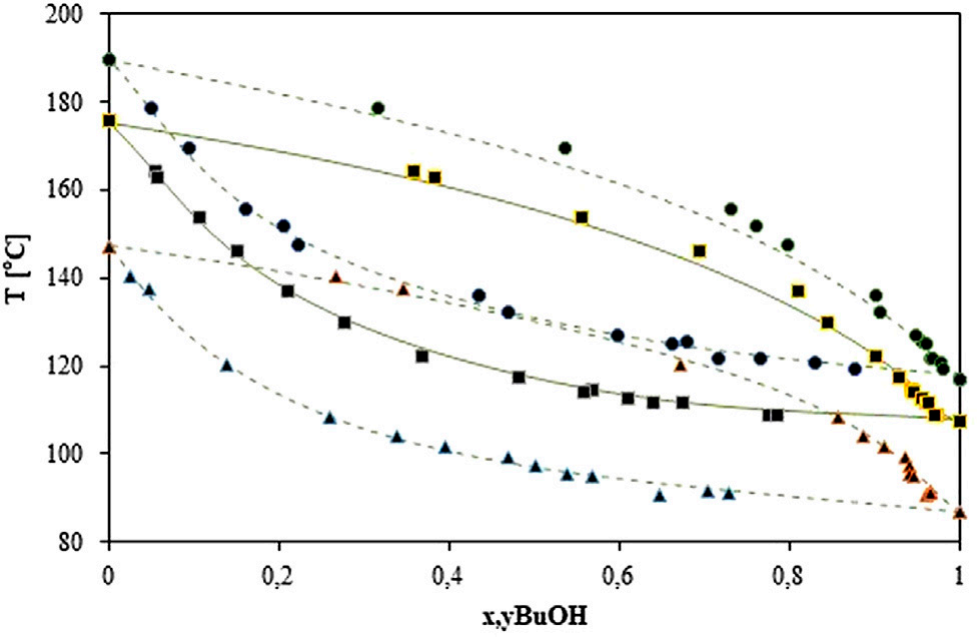
T−x−y diagram for the system butanol–butyl glycolate measured experimentally at 300 mbar (▲), 700 mbar (■), and 1013.25 mbar (●); and calculated by NRTL adjusted (---).

The NRTL binary interaction parameters were calculated at 300.00, 700.00, and 1013.25 mbar, and are summarized in Supplementary Table S4. The VLE predicted values and the VLE measured experimentally are compared and shown in Figure 4. It is observed that for the three pressures, it is a good agreement between the predicted and the measured equilibrium values. The total differences between the values predicted by the NRTL model and the experimental data are 1.1%.

#### 3.1.3 Liquid–Liquid Equilibrium of Butyl Glycolate–Water

**FIGURE 5:**
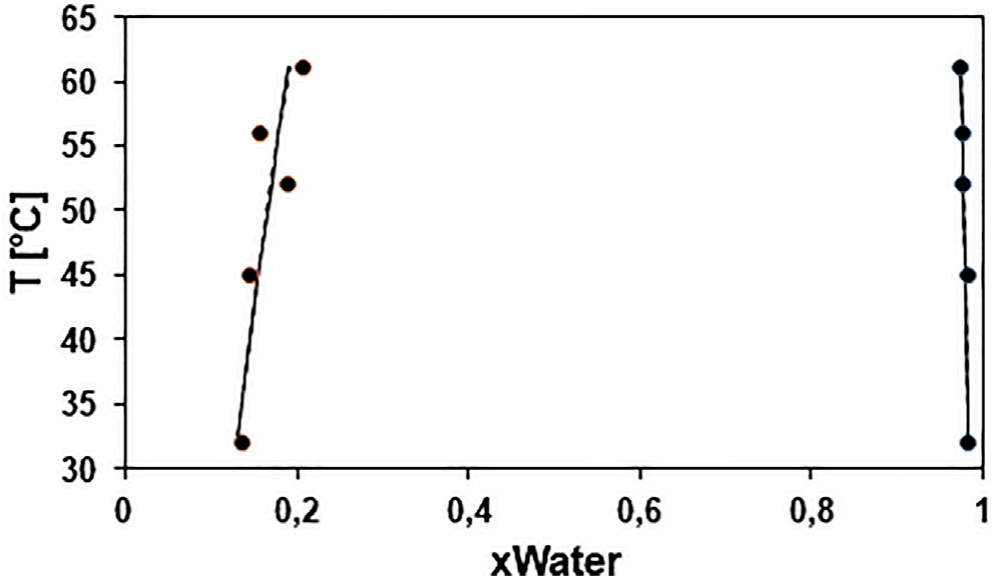
T−x−y diagram for the system BG + W at 1013.25 mbar. Exp (●), NRTL (-).

Figure 5 shows the LLE data of the BG-W system within a temperature range of 32.05°C–61.15°C, at atmospheric pressure. From these data and using Aspen Plus V10 regression tool, the binary parameters for the NRTL and UNIQUAC models were determined. The predicted values using the two models are also presented in Figure 5. It is observed both models can accurately predict the LL equilibria. For both models, the root mean square error (RMSE) percentage for temperature prediction was of 0.4%. The equilibria were more accurately predicted at high water (W) compositions, rather than at high butyl glycolate (BG) compositions.

The binary interaction parameters for both models are summarized in Supplementary Table S4. For NRTL model, α_ij_ was set to 0.2 (Toikka et al., 2018).

#### 3.1.4 Solid–Liquid Equilibria

The solid–liquid equilibrium is widely used in the literature to determine the activity coefficients of a solute (Negadi et al., 2006; Gutiérrez et al., 2019). For GA as solute, the equilibrium equation can be expressed as follows:

$$ln\left(a_{GA}\right)=\ln\left(x_{GA}\gamma_{GA}\right)=\frac{\text{Δ}H_{fus}}{R}\left[\frac{1}{T_{fus}}-\frac{1}{T}\right]+\left(\frac{1}{RT}\right)\int_{T}^{T_{fus}}\left(C_{pL}-C_{pS}\right)dT\;-\left(\frac{1}{R}\right)\int_{T}^{T_{fus}}\frac{\left(C_{pL}-C_{pS}\right)dT}{T}dT-\lambda_{PT}$$ (11)

$a_{GA}$ , $x_{GA}$ , and $\;\gamma_{GA}$ are respectively the activity, molar fraction, and activity coefficient of glycolic acid in saturated solution. $\text{Δ}H_{fus}$ is the fusion enthalpy, $C_{pL}$ and $C_{pS}$ are the heat capacities of GA in the liquid and solid state, respectively; and T and $T_{fus}$ are the temperatures of the system and melting point of GA, respectively.

**FIGURE 6:**
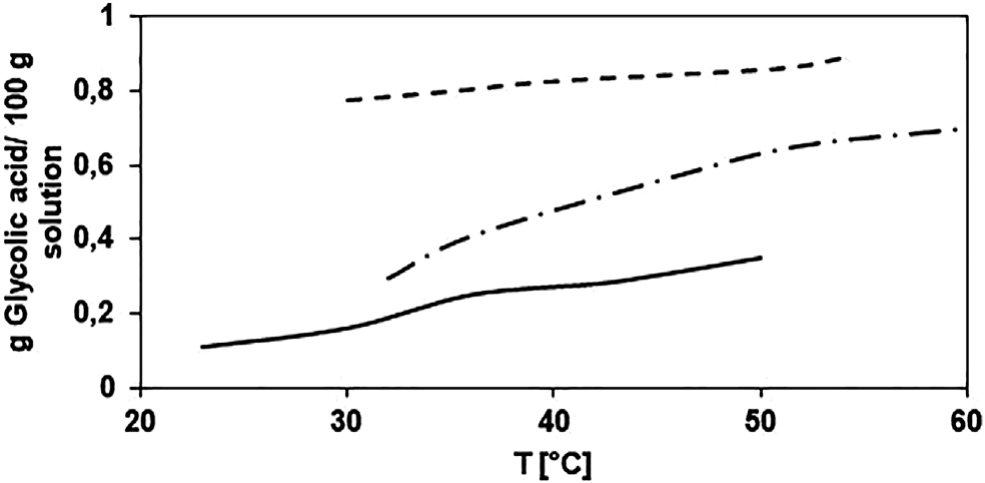
Glycolic acid solubility in water (- - -), butyl glycolate (-- - --), and butanol (▬).

The activity coefficients for GA $\left(\gamma_{GA}\right)$ were calculated from the experimental solubility curves ( $T,\;x_{GA}$ ), obtained in water, butyl glycolate, and butanol (Figure 6). For the calculation; the melting point and enthalpy of fusion of GA were taken from the literature (Emel’yanenko et al., 2010), and the heat capacities of liquid and solid were calculated using the Aspen Plus V10 estimation tool. The values and expressions are summarized in Supplementary Table S4.

The binary parameters were fitted for the NRTL model by minimizing the difference between the calculated activity coefficients using the binary NRTL parameters and the experimental values determined using Eq. 8. The minimization was performed using the MATLAB 9.4 tool, and the results are presented in Supplementary Table S4.

### 3.2 Kinetic Studies

#### 3.2.1 Equilibrium Measurement

The equilibrium constants $\left(K_{eq}\right)$ were determined using Eq. 12, where $K_{x}$ represents the ratio of the molar fractions of the mixture at the equilibrium, and $K_{\gamma }$ represents the relation of the activity coefficients calculated at the same conditions. The activity coefficients of the components of the reaction mixture were calculated by the NRTL method using the binary parameter determined in the thermodynamic study (Supplementary Table S4). The equilibrium constants are presented in Table 2.

$$K_{Eq}=K_{x}K_{\gamma }=\frac{\left(x_{Eq_{C}}*x_{Eq_{D}}\right)}{\left(x_{Eq_{A}}*x_{Eq_{B}}\right)}\;\frac{\left(\gamma_{Eq_{C}}*\gamma_{Eq_{D}}\right)}{\left(\gamma_{Eq_{A}}*\gamma_{Eq_{B}}\right)}$$ (12)

The comparison between the values of K_x_ and K_eq_ concludes that at low temperatures, the system tends to follow an ideal behavior. Indeed, by comparing Eqs 13, 14, which represent the evolution of K_eq_ and K_x_ as a function of temperature, we notice that K_eq_ is equal to K_x_ for a temperature lower than or equal to 45°C. However, when the temperature increases, there is a significant difference between the ideal and nonideal system. By including the effect of nonideality in the calculation of the equilibrium constant, an increase in the total value is observed. These results are in agreement with those presented by Orjuela et al. (2012). They found, for the succinic acid and ethanol system, an increase in a factor of four for the equilibrium constant for a temperature range of 70°C–120°C; in this study, the increase in factors was found to be between 1 and 1.5. However, they consider that for their reaction, the effect of temperature was negligible. In the present study, the effect of temperature was considered in order to reduce possible sources of error as much as possible and to achieve the best possible adjustment. Considering the equilibrium constant, as a function of the activities, the curve corresponded to an endothermic reaction and the standard enthalpy of reaction can be calculated from the Van’t Hoff equation (see Eq. 13). In the literature, there are no works similar to our case study; the closest result is the value reporter by Xu et al. (2012) who worked on the hydrolysis of methyl glycolate (reverse reaction). They found a standard reaction enthalpy of −15.52 kJ mol^−1^, which is in agreement with our calculated value.

$$\ln K_{eq}=-\frac{2858.5}{T}+9.41$$ (13)

$$\ln K_{x}=-\frac{1125.7}{T}+3.96$$ (14)

**TABLE 2:** Equilibrium constants of glycolic acid esterification with butanol.

Temp (°C)	Kx	Keq
50	1.60	1.73
60	1.80	2.39
70	1.96	2.90

#### 3.2.2 Catalyzed Reaction

##### 3.2.2.1 Diffusional Limitations

The study to determine diffusion limitations was performed for the esterification reaction of GA and butanol. Different stirring rates were evaluated to determine the effect of external mass transfer resistance. At the same reaction time, the conversion rate remained constant despite the change in the stirring rate, suggesting that the external mass transfer resistance is not the speed-controlling step (Supplementary Figure S1). This result agrees with that of different works carried out with NAFION NR50^®^ (Lopez et al., 2007) or Amberlyst 36 (Akyalçın, 2017).

The effect of intra-particle diffusion in the reaction was studied for the Amberlyst 36. Two different particle sizes were screened, between 250 and 500 μm and greater than 500 μm. Through the experiments, it was observed that there were no evident differences in reaction rates with change in particle sizes, which shows that internal resistance to mass transfer can be neglected. This work could not be done with Nafion^®^ NR50, which has a larger nominal size than most ion-exchange resins and cannot be ground. For each experiment, the Weisz modulus was calculated (Supplementary Table S6), taking into account the effective diffusivity of GA since it is the limiting reagent.

$$\varphi_{su}^{\prime }=\frac{\bar{r_{p}}L^{2}}{D_{eAG}C_{AG}^{su}}$$ (15)

where $\bar{r_{p}}$ represents the apparent kinetic rate; L, the characteristic length of catalyst equal to volume and surface ratio; D_eAG_, the effective diffusion coefficient of glycolic acid; and C^su^_AG_, the concentration of glycolic acid on the surface of the catalyst.

For each experiment, the Weisz modulus was less than 0.1. These results are in agreement with those of the extensive literature, which state that external diffusion and intra-particle resistances are usually negligible for most reactions catalyzed by Amberlyst-type resins (Orjuela et al., 2012; Nguyen et al., 2018; Toor et al., 2011; Pappu et al., 2013). This allows the kinetic study to be carried out with the certainty of working in a kinetic regime.

##### 3.2.2.2 Effect of the Catalyst

**FIGURE 7:**
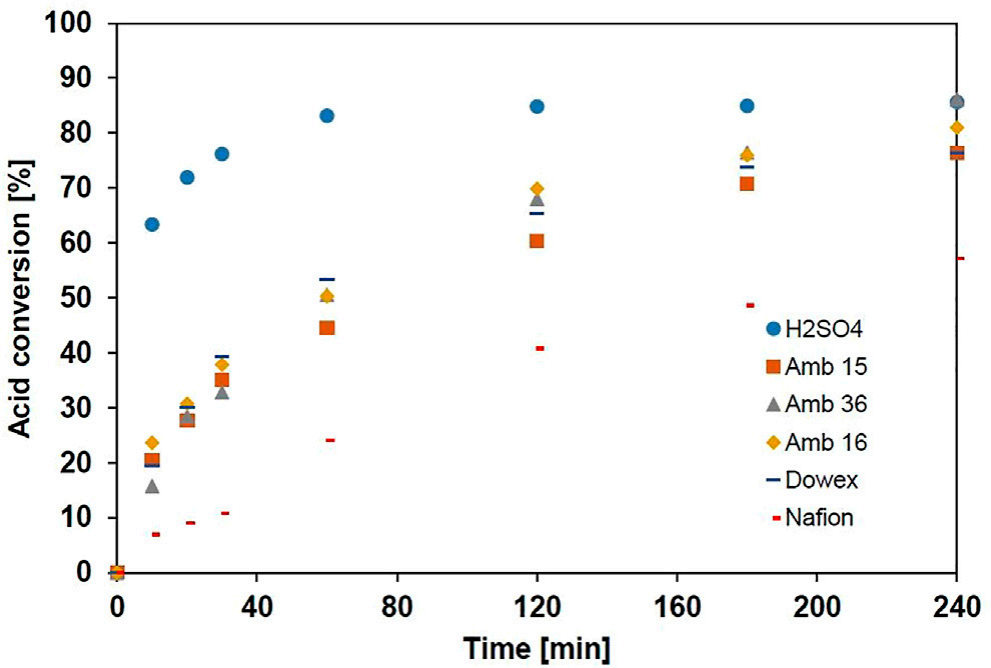
Effect of catalyst type on glycolic acid conversion rate with butanol.

Figure 7 compares the evolution of the glycolic acid conversion rate using different commercial ion-exchange resins as catalysts. The test performed using H_2_SO_4_ under the same conditions is also presented in Figure 7 as a benchmark. All resins showed similar catalytic behavior, with high activities and slower kinetics compared with H_2_SO_4_. After 240 min, only the Amberlyst 36 achieved a conversion rate equal to the one obtained with H_2_SO_4_ (i.e., 86%). Amberlyst 16 also showed high activity with a conversion rate of only 5% lower compared to that obtained with the H_2_SO_4_. In this case, it is noticed that the activity does not depend on the morphology of the catalyst, but it can be related to its ion-exchange capacity. Dowex and Amberlyst15 despite having a capacity superior or equal to 4.7 eq.kg^−1^ have similar conversions, both having 10 activity points less than Amberlyst 36. Amberlyst 36 has been widely used for esterification reactions due to its advantages in terms of catalytic activity and thermal stability, as already mentioned (Pappu et al., 2013; Akyalçın, 2017; Tsai et al., 2011; de Aguiar et al., 2017). Liu et al. (2006) have shown that in order for Nafion resin to have a similar activity to H_2_SO_4_, its polymeric structure must be modified; the authors also found that the activity of this catalyst is strongly inhibited by the presence of water. This does not seem to be the case here, which is why this resin will be studied in more detail.

##### 3.2.2.3 Kinetic Model

For the kinetic study, the data were adjusted using a pseudo-homogeneous (PH) model and adsorption-based models as presented in the literature (see Table 3). Among the authors who used the PH model, Steinigeweg and Gmehling (2002) demonstrated that this model is sufficient to describe the profiles of the reactive distillation columns if there are small or medium amounts of water in the system. Orjuela et al. (2012) also demonstrated that the PH model can describe the esterification reaction between succinic acid and ethanol; they also considered the dehydration reaction of ethanol, a secondary reaction in the studied conditions. The model most used in the literature to describe the kinetic behavior of the esterification reaction in the presence of heterogeneous catalysts is the Langmuir–Hinshelwood (LH) model; this model considers that all compounds are adsorbed on the surface of the catalyst (Gangadwala et al., 2003). Finally, another model also mentioned and studied in the literature is the Eley–Rideal (ER) model. This model considers that the esterification reaction occurs between the adsorbed alcohol and non-adsorbed acid to obtain the non-absorbed ester and adsorbed water molecules (Ju et al., 2011).

**TABLE 3:** Kinetic models for esterification reaction.

Model	Kinetic law	
pseudo-homogeneous (PH)	The reaction takes place as if it were a homogeneous system	$k^{+}\left(a_{GA}a_{BuOH}-\frac{a_{BG}a_{H_{2O}}}{K_{eq}}\right)$
Eley–Rideal (ER)	Only one reactant and one product are found as adsorbed species	$\frac{k^{+}\left(a_{GA}a_{BuOH}-\frac{a_{BG}a_{H_{2O}}}{K_{eq}}\right)}{\left(1+K_{GA}a_{GA}+K_{BG}a_{BG}\right)}$
Langmuir–Hinshelwood (LH)	All reactants and products are present as adsorbed species	$\frac{k^{+}\left(a_{GA}a_{BuOH}-\frac{a_{BG}a_{H_{2O}}}{K_{eq}}\right)}{{\left(1+K_{GA}a_{GA}+K_{BuOH}\;a_{BuOH}+K_{BG}a_{BG}+K_{H_{2}O}a_{H_{2}O}\right)}^{2}}$

The mass balance is a system of ordinary differential equations and is solved using an ordinary differential equation solver ode45 in MATLAB 9.4^∗^. The fourth-order Runge–Kutta method was used to numerically integrate the kinetic model. The optimization of the kinetic parameters was performed by minimizing the residual sum of squares (SRS) between experimental ( $x_{exp}$ ) and calculated ( $x_{cal}$ ) species mole fractions using the following objective function, represented in a general way in the equation:

$$SRS=\frac{1}{n}\sum_{Allsamples}^{Nc}{\left(x_{exp}-x_{cal}\right)}^{2}$$ (16)

Where n is the number of experimental samples taken from the batch reactors in all experiments performed, and NC is the number of components considered in each sample.

To confirm that the parameters found correspond to the global optimum, the “Multistart” function was used, generating different combinations of initial points. For the adjustment of the variables, the thermodynamic consistency was considered, forcing positive values of the kinetic and adsorption constants.

As the last stage of verification, the absolute and relative error or mean relative deviation of the optimized constants was determined as presented in the following equations:

$$E_{Abs}=\;\frac{1}{n}\sum_{Allsamples}^{Nc}\left|x_{exp}-x_{cal}\right|$$ (17)

$$E_{Rel}=\frac{100*1}{n}\sum_{Allsamples}^{Nc}\frac{\left|x_{exp}-x_{cal}\right|}{x_{exp}}$$ (18)

**FIGURE 8:**
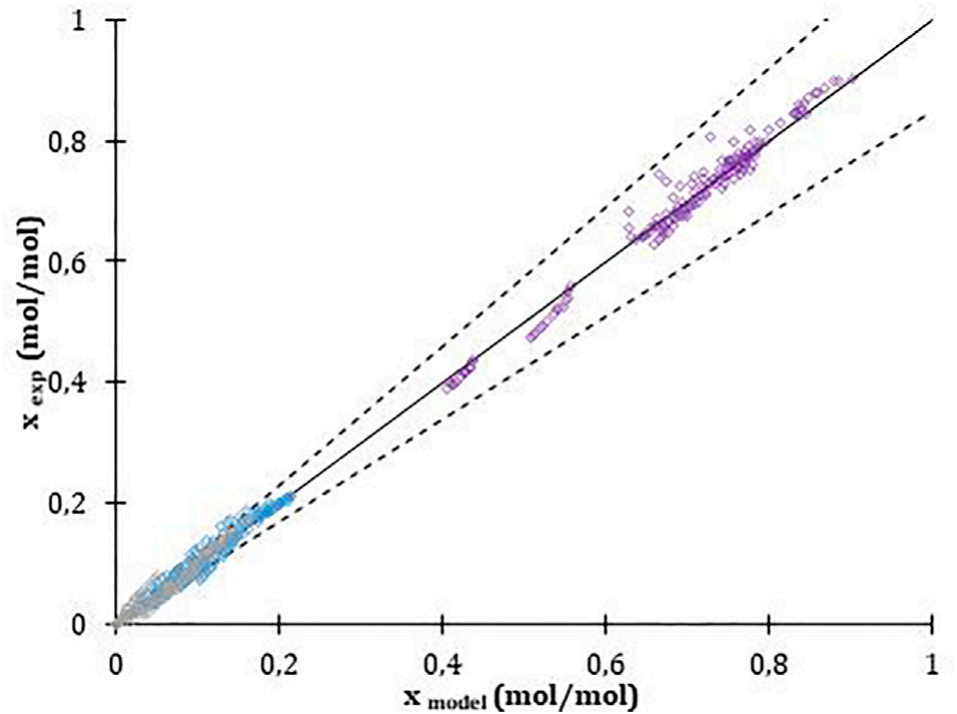
Parity diagram for esterification of GA with butanol and Nafion NR^®^ as catalyst.

Kinetic parameters were determined for two catalysts (Amberlyst 36 and Nafion NR50^®^) with 400 experimental data in each case (10 experiments * 10 samples * 4 compounds). The obtained parameters for each reaction with the three tested models are listed in Supplementary Table S5. Figure 8 presents an example of the parity diagram between the experimental molar fraction and the modeled ones using the parameters given in Supplementary Table S5. A very good fit is obtained between the experimental compositions and the calculated ones with values within an error of 10%.

The results of the adjustment show that the error decreases when the adsorption-based models are considered, although the difference between the results obtained using the PH model and adsorption-based models varies by a maximum of 1.4%. This coincides with the conclusion of studies carried out by Steinigeweg and Gmehling (2002) and Orjuela et al. (2012) in which it is shown that the PH model can describe with great precision this type of reaction in the presence of ion-exchange resins such as Amberlyst type.

Considering the models that represent the adsorption phenomenon, there is a slight increase in the activation energy from 53 to 56 kJ.mol^−1^. The activation energy of the esterification reaction using Amberlyst 36 is 56 kJ.mol^−1^; the value is similar regardless of the model used for the adjustment. However, when considering the adsorption-based models, the activation energy increases, although on this particular reaction, no information is available in the literature, the values obtained are within the range of values using the same type of catalyst, type of alcohol, and primary and carboxylic acids such as carbon numbers between C2 and C4. Steinigeweg and Gmehling (2002) reported the activation energy value of 56.65 kJ mol^−1^ for the system between acetic acid and butanol in the presence of Amberlyst 15 as catalyst. This value could be compared with the values obtained in this study since in both cases it is a carboxylic acid with two carbons. Additionally, the glycolic acid contains an -OH group. No secondary reaction associated with the presence of this -OH group was observed under the studied conditions. It is worth noting that the activation energy increases by approximately 20% when switching from a homogeneous catalyst to a heterogeneous catalyst.

The best fit of the experimental data was achieved using the ER model; the adjustment of the experimental data demonstrates that it is only the butanol, the only molecule that strongly adsorbed from the reagents, and the water molecule in the products. However, the results obtained with the pH model and the LH model are also accurate.

### 3.3 Reactive Distillation Experiments and Simulation

#### 3.3.1 Reactive Residue Map

The reactive residue curve map obtained shows that there not exist reactive azeotropes and the heteroazeotrope between butanol and water and the azeotrope between GA and BG remains despite the reaction (Supplementary Figure S2). Pure n-butanol, pure water, and azeotrope between GA and ester are saddle nodes, so they cannot be obtained by reactive distillation unless a second feed is added. On the contrary, GA and BG are stable nodes and the water/n-butanol azeotrope is an unstable node. Finally, there is a distillation boundary between the unstable node (the water/n-butanol heteroazeotrope) and the saddle point (GA/BG azeotrope). Thus, it can be concluded that with a rich n-butanol reacting mixture, a reactive separation process in order to produce pure BG is feasible. Finally, it is mainly the position of the distillation frontier that is impacted by the increase in pressure (presented in Supplementary Figure S2). Thus, by decreasing the pressure, the operating range for ester production is also reduced.

#### 3.3.2 Repeatability, Steady State, and Mass Balance

**FIGURE 9:**
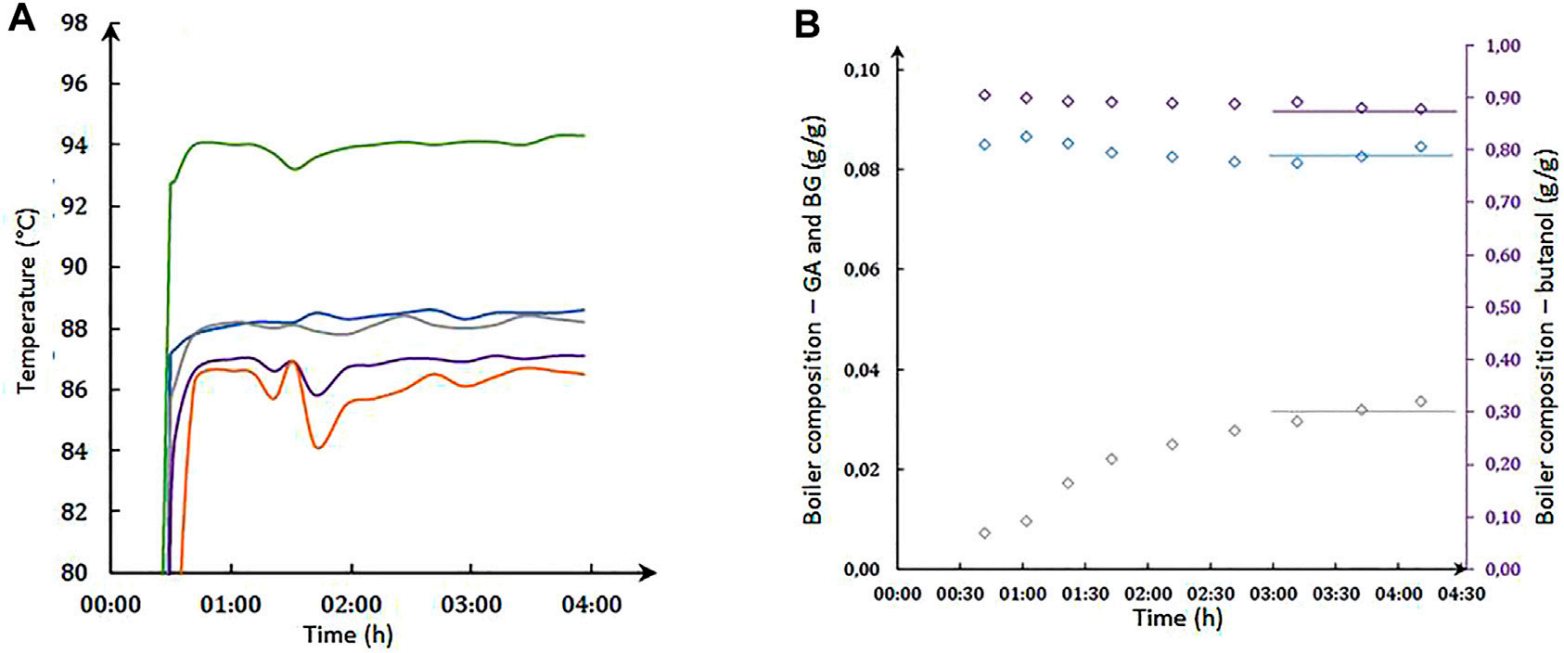
Steady-state establishment with feed of 0.5 kg h^−1^, *p* = 380 mbar, and R = 1. **(A)** Temperature in column T_1_ (boiler) (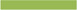), T_2_ (column bottom) (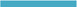), T_3_ (mid-column) (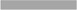), T_4_ (column top) (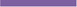), and T_5_ (condenser) (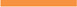) versus time; **(B)** boiler composition in BA, GB, and butanol versus time.

Figure 9 shows the composition of the residue and the temperatures along the column versus the time. Steady state is achieved after 4 h. To limit transient regimes, the start-up strategy was adjusted using the composition of the previous experiment in the boiler. For each experiment carried out, the carbon balance is verified to within 0.1%, and it can be stated that there are no carbon by-products detected during the esterification between GA and n-butanol. Among other things, polycondensation reaction does not take place. Experiment with 8 g of Nafion^®^ N50 catalyst, a feed rate of 0.66 kg h^−1^ with 1:10 acid/butanol mass ratio, and a reflux of 1 was performed twice in order to verify the repeatability of the experiments and the catalytic performance. As shown in Table 1, the conversion rates are similar, as well as the flow rates and residue compositions. The flow rate in distillate differs, possibly due to heat losses as the column head is not jacketed. Experiments can be considered repeatable. The catalyst used in these experiments will have been stable over more than 12 h of operation.

#### 3.3.3 Effect of Operating Parameters

To study the effect of reflux ratio in the process, this was changed keeping constant the rest of parameters. The ratios studied were 0, 0.5, 1, and 5. There is no significant change in the molar composition in the distillate. Moreover, the recovery rate of the ester is 100% at the residue in all the experiments, which is particularly interesting from the point of view of recovery of the acid, for example, by hydrolysis of the ester. At high reflux ratios, the reactants are separated too effectively from each other, which reduces the reaction rate and causes the less conversion rate of glycolic acid. If a high reflux ratio is used, ethanol leaves the column with the distillate. For optimum reaction conditions, the concentrations of both reactants must be high in the reaction zone. The highest conversion rate of glycolic acid is 33%, obtained at zero reflux. The column behaves like a reactive stripping column, similar to the result reported by Fields et al. (2008).

**FIGURE 10:**
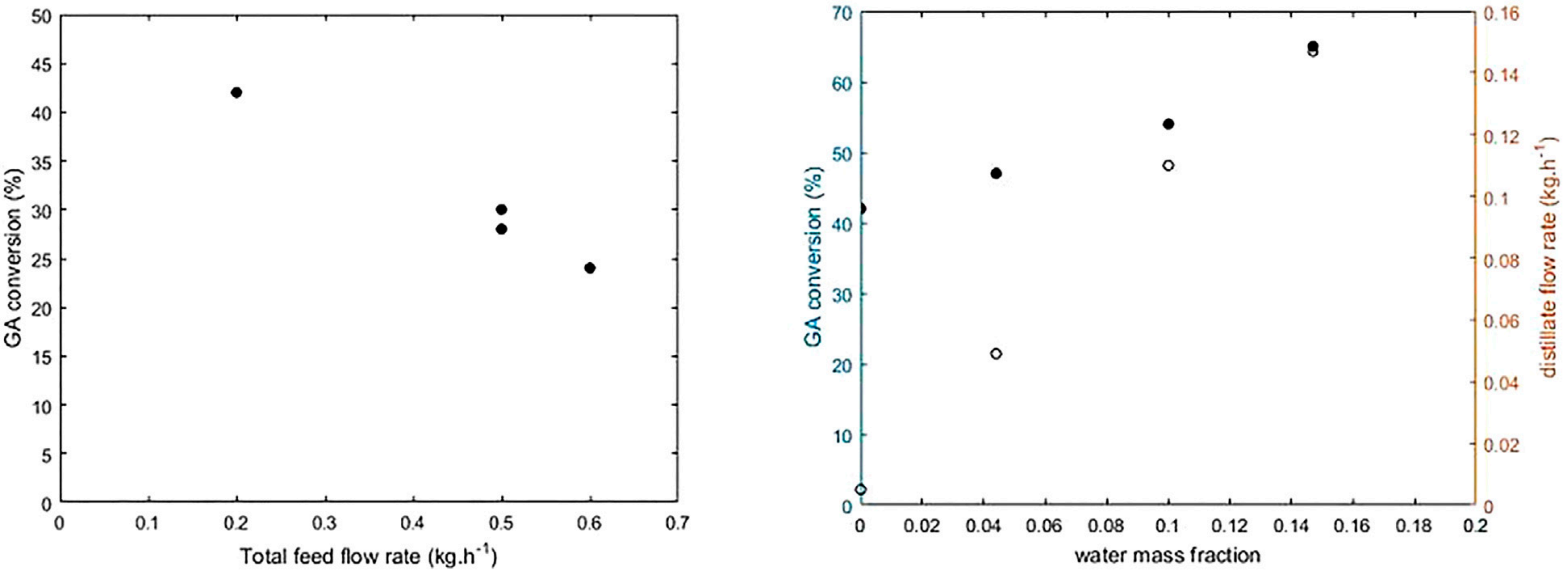
Conversion rate of glycolic acid versus total feed flow rate **(A)** versus water mass fraction **(B)** (R = 1, m_cat_ = 22 g, *p* = 380 mbar and total feed rate 0.2 kg h^−1^).

Figure 10 shows the conversion rate of GA versus total feed flow rate. The reboiler temperature is 90°C, the feed mass ratio is 1:10, and the reflux ratio is 1. The conversion rate decreases when the total feed rate is increased from 0.2 to 0.6 kg h^−1^, mostly due to less residence time of reactants in the reactive zone. At the highest feed flow rate, it is possible to increase the conversion rate by increasing the amount of catalyst and therefore the reactive zone. However, by having twice the contact time between liquid and solid, that is, by increasing the catalyst mass from 10 to 20 g, the conversion rate only increases by 26%. This result suggests that there is external and/or internal diffusional limitation in the column. Moreover, inadequate column hydrodynamics can lead to partial wetting of the catalyst. With the maximum amount of catalyst (i.e., a whole column section), a conversion rate of 65% is achieved, which is still below the conversion rate at thermodynamic equilibrium (85%).

Figure 10 shows the increase in the conversion rate of GA and the distillate flow rate with the mass ratio of water in feed flow rate. The GA conversion rate increases with an increase of water in feed. The excess water causes more vapor generation in the reboiler, which leads to high vapor flow rate in the reactive zone and an increase in the distillate flow rate. On the contrary, when the vapor flow rate is higher, the contact time of liquid on solid catalyst is lower. Hence, the conversion rate should decrease. However, the estimation of the external resistance fraction (Eq. 19) and the Weisz modulus (Eq. 15) showed that by increasing the amount of water in the liquid phase, the diffusional limitations decrease (Table 4). Although the chemical regime is not yet achieved, under the conditions of our experiments the presence of water in the feed is beneficial for the recovery of GA.

$$f_{ex}=\frac{\bar{r_{p}}L}{k_{D_{AG}}C_{AGex}}$$ (19)

**TABLE 4:** Mass transfer resistance data vs. %.mt H_2_O.

x_H2O_	f_ex_	ϕ′_su_	$\eta =\eta_{su}\left(1-f_{ex}\right)$ (%)
0	0.447	21.1	3
0.14	0.257	4.7	26
0.28	0.137	3.1	28
0.36	0.107	1.9	47

#### 3.3.4 Simulation

The parameters of the simulations were confirmed with the experimental results for GA conversion (Supplementary Table S7). The relative difference between simulations and experimental results is less than 6% for the conversion rate (e.g., 27% by simulation versus 25%) and recovery rate of the ester to residue (e.g., 99% versus 100%). Nevertheless, the ester purity is lower in simulations (by 15% on average, e.g., 4.9% versus 5.7%), which can be explained by a lower reboiler heat in simulations. Indeed, this results in a lower vapor rate and a less efficient separation. Moreover, the ester is highly diluted so the purity is always under 10%. A small deviation on the moles recovered in the boiler obtained by simulation can lead to a large deviation between simulation and experimental data on this parameter.

**FIGURE 11:**
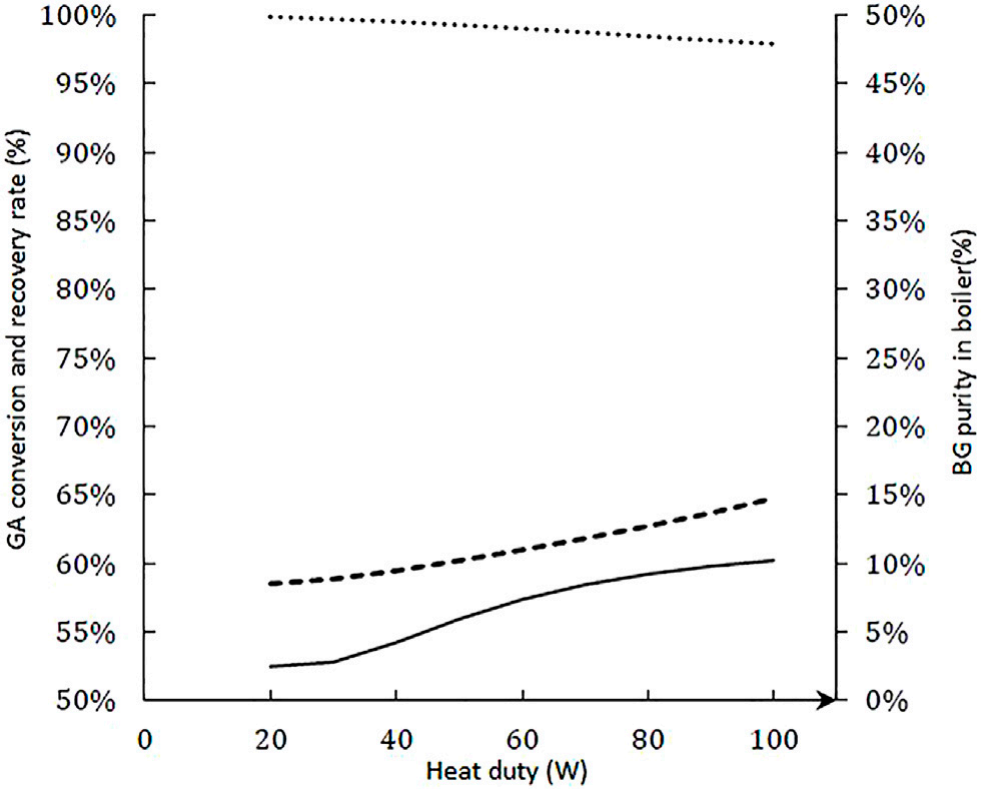
Conversion rate of glycolic acid (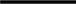), purity (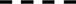), and recovery rate of butyl glycolate in residue (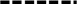) versus the boiler heat.

Figure 11 shows the conversion rate of GA, purity, and recovery rate of BG in residue versus the boiler heat. This heat duty has a low impact on the conversion rate. However, by increasing the heating, the BG purity increases while keeping a recovery rate above 98%. However, it is important to note that increasing the heat duty leads to a high flow of steam and increases the risk of clogging the column. This parameter must therefore be controlled. To avoid clogging, to favor the gas/liquid flow, and to keep the catalytic efficiency, it would be possible to use a reactive divide wall column. The addition of a partial wall inside the column or and internal tube where the steam flows inside the column allows the purity of the products recovered at the outlet of the unit (Weinfeld et al., 2018; Von Harbou et al., 2011). Finally, a fully reactive column was compared with the initial configuration (i.e., 2 separation stages and just one reactive stage). A conversion rate of 80% and a recovery rate of 99.5% of the ester to the residue were obtained versus 68% and 99.4%, respectively, for the initial simulation. Therefore, there is no impact on the liquid phase composition of the presence of a pure separation zone. This result confirms that the column behaves as a reactive stripping column. The influence of the position of the feed on the conversion rate was studied by simulation. The reactive section is number 3 (Section 4 is the boiler). The conversion rate increases from 39% to 60% depending on whether the feed is positioned at stage 1 or 3, respectively. On the contrary, the recovery rate of ester to residue is 100% regardless of configuration. Finally, a feed in the boiler leads to a decrease in the ester recovery rate. Finally, the purity of the ester to residue is the highest (17%) when the feed is located at the top of the distillation column. Indeed, most of the butanol is recovered at the distillate in this configuration. The concentration of ester decreases strongly with a boiler feed, due to the decrease in the conversion rate. The associated simulations are provided in Supplementary Figure S3.

## 4 Conclusion

In this study, a thermodynamic model based on the NRTL model was developed and validated by fitted experimental data. A screening of catalysts was carried out. It was shown that Amberlyst 36 was the most efficient. Nafion resins were also chosen for their ease of use in the distillation columns. The kinetic study of the esterification in the presence of homogeneous and two heterogeneous catalysis was also carried out. It was concluded that the heterogeneous reaction can be accurately described either by a pseudo-homogeneous model or the Langmuir–Hinshelwood (L-H) adsorption model. A parametric study of this esterification in a reactive distillation pilot showed no significant effect of reflux ratios, but the conversion rate of GA increases with the residence time in the column. The results obtained with a different mass of catalyst suggest that there is external and/or internal diffusional limitation in the column. With the maximum amount of catalyst (i.e., a whole column section), a conversion rate of 65% is achieved, which is still below the conversion rate at thermodynamic equilibrium (85%). Based on the kinetic and thermodynamic models developed, the simulation of the reactive distillation column with ProSim Plus showed that to increase the ester yield, operating at a low feed rate with reactive stripping was sufficient.

## Data Availability

The original contributions presented in the study are included in the article/Supplementary Material; further inquiries can be directed to the corresponding author.

## Glossary

**a**: activity
**c**: concentration, mol l^−1^
**c_p_**: heat capacity, J.mol^−1^ K^−1^
**D_e_**: effective diffusion coefficient, m^2^ s^−1^
**E_abs_**: absolute error, %
**E_rel_**: relative error, %
**f_ex_**: external fraction resistance
$\Delta H_{\mathrm{fus}}$: fusion enthalpy, J.mol^−1^
**K_DAG_**: convection coefficient of glycolic acid, m s^−1^
**K_eq_**: equilibrium constant
**K_x_**: ratio of the molar fractions of the mixture
**L**: volume-to-surface ratio, m
**n**: number of experimental samples
**P**: total pressure of the system, bar
**P°**: vapor pressure, bar
**R**: ideal gas constant, J.mol^−1^ K^−1^
$\bar{r_{p}}$: apparent kinetic rate, mol.s^−1^ kg^−1^
**SRS**: residual sum of squares
**T**: temperature, °C or K
**V**: vapor flow, mol.s^−1^
**X_i_**: transformed molar faction of compound i in liquid phase
**x_i_**: molar fraction of compound I in liquid phase
**Y_i_**: transformed molar fraction of compound I in vapor phase
**y_i_**: molar fraction of compound I in vapor phase
$\nu_{i}$: algebraic stoichiometric coefficient
**φ′**: Weisz modulus
**φ**: fugacity coefficient
**γ**: activity coefficient
**σ^j^**: standard deviation of j parameter
**BuOH**: butanol
**BG**: butyl glycolate
**calc**: calculated
**ex**: external to the catalyst
**exp**: experimental
**fus**: melting point
**GA**: glycolic acid
**i**: component i
**l**: liquid
**s**: solid
**su**: surface of the catalyst
