# Supplementary material for: Reactive Distillation of Glycolic Acid Using Heterogeneous Catalysts: Experimental Studies and Process Simulation
Source: Front Chem. 2022 Jun 15;10:909380. doi: 10.3389/fchem.2022.909380 (PMC9240659; doi:10.3389/fchem.2022.909380)

**Table S1. Description of Chemicals Used in Experiments**

| **Chemical name  ()^a^** | **CASRN** | **Suppliers** | **Purity %^b^** | **Analysis**  **method** |
| --- | --- | --- | --- | --- |
| **Butyl glycolate (BG)** | 7397-62-8 | Sigma-Aldrich | >95 (99)^c^ | GC^d^ |
| **n-Butanol  (BuOH)** | 71-36-3 | Alpha Aesar | 99.5 | GC^d^ |
| **Water  (W)** | 7732-18-5 | HPLC grade |  |  |
| **Glycolic acid (GA)** | 79-14-1 | Alpha Aesar | 98 |  |
| **Sulfuric acid** | 7664-93-9 | Sigma-Aldrich | 95- 98 |  |
| **Acetonitrile (ACN)** | 75-05-8 | HPLC grade |  |  |

*^a^ Nomenclature used in the article, ^b^Wt.%;  ^c^ After vacuums distillation^. d^ Gas chromatography*

**Table S2: experimental datat for BG vapor pressure**

| Pressure (mbar) | Temperature (°C) |
| --- | --- |
| 1 | 30.05 |
| 1 | 30.1 |
| 2 | 40.14 |
| 4 | 50.09 |
| 6 | 60.03 |
| 11 | 70.12 |
| 18 | 80.13 |
| 31 | 90.19 |
| 49 | 100.6 |
| 79 | 110.74 |
| 100 | 119.38 |
| 200 | 136.09 |
| 300 | 146.99 |
| 400 | 156.78 |
| 500 | 164.52 |
| 600 | 169.42 |
| 700 | 175.42 |
| 800 | 179.23 |
| 900 | 185.00 |
| 1013.25 | 189.39 |

**Table S3: experimental data of the VLE BG + BuOH**

T-x-y data for the system BG + BuOH at 1013.25 mbar

| **T [°C]** | **T [K]** | **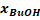** | **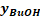** | **P° (bar)** | **γ** |
| --- | --- | --- | --- | --- | --- |
| **189.39** | 462.39 | 0.000001 | 0 | 2.405 |  |
| **178.49** | 451.49 | 0.049 | 0.317 | 2.154 | 3.044 |
| **169.34** | 442.34 | 0.094 | 0.536 | 1.952 | 2.960 |
| **155.69** | 428.69 | 0.16 | 0.733 | 1.668 | 2.783 |
| **151.79** | 424.79 | 0.204 | 0.761 | 1.591 | 2.376 |
| **147.54** | 420.54 | 0.222 | 0.8 | 1.509 | 2.420 |
| **136.24** | 409.24 | 0.435 | 0.902 | 1.302 | 1.614 |
| **135.04** | 408.04 | 0.491 | 0.901 | 1.280 | 1.452 |
| **133.58** | 406.58 | 0.483 | 0.918 | 1.255 | 1.534 |
| **132.37** | 405.37 | 0.47 | 0.909 | 1.234 | 1.588 |
| **127.09** | 400.09 | 0.598 | 0.948 | 1.145 | 1.403 |
| **125.76** | 398.76 | 0.679 | 0.957 | 1.123 | 1.271 |
| **125.27** | 398.27 | 0.661 | 0.961 | 1.115 | 1.321 |
| **124.36** | 397.36 | 0.716 | 0.963 | 1.100 | 1.238 |
| **123.39** | 396.39 | 0.717 | 0.954 | 1.085 | 1.243 |
| **121.69** | 394.69 | 0.718 | 0.966 | 1.058 | 1.289 |
| **121.62** | 394.62 | 0.765 | 0.969 | 1.057 | 1.215 |
| **120.75** | 393.75 | 0.83 | 0.979 | 1.043 | 1.146 |
| **119.21** | 392.21 | 0.879 | 0.98 | 1.019 | 1.109 |
| **117.02** | 390.02 | 1 | 1 | 0.985 | 1.029 |

**
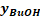

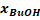
**T-x-y data for the system BG + BuOH at 700 mbar

| **T [°C]** | **T [K]** |  |  | **P° (bar)** | **γ** |
| --- | --- | --- | --- | --- | --- |
| **175.85** | 448.85 | 0 | 0 | 2.094 |  |
| **164.42** | 437.42 | 0.054 | 0.357 | 1.847 | 2.505 |
| **162.71** | 435.71 | 0.057 | 0.382 | 1.811 | 2.590 |
| **153.93** | 426.93 | 0.106 | 0.555 | 1.633 | 2.244 |
| **145.86** | 418.86 | 0.151 | 0.693 | 1.477 | 2.175 |
| **137.09** | 410.09 | 0.21 | 0.811 | 1.317 | 2.053 |
| **130.01** | 403.01 | 0.277 | 0.844 | 1.194 | 1.786 |
| **122.23** | 395.23 | 0.369 | 0.901 | 1.066 | 1.603 |
| **120.04** | 393.04 | 0.495 | 0.929 | 1.032 | 1.273 |
| **119.44** | 392.44 | 0.49 | 0.926 | 1.022 | 1.294 |
| **117.34** | 390.34 | 0.482 | 0.93 | 0.990 | 1.364 |
| **114.5** | 387.5 | 0.568 | 0.943 | 0.947 | 1.227 |
| **114.13** | 387.13 | 0.559 | 0.945 | 0.942 | 1.257 |
| **112.52** | 385.52 | 0.611 | 0.957 | 0.918 | 1.194 |
| **111.76** | 384.76 | 0.674 | 0.962 | 0.907 | 1.102 |
| **111.53** | 384.53 | 0.64 | 0.964 | 0.903 | 1.167 |
| **108.9** | 381.9 | 0.775 | 0.974 | 0.866 | 1.016 |
| **108.64** | 381.64 | 0.785 | 0.972 | 0.862 | 1.006 |

T-x-y data for the system BG + BuOH at 300 mbar

| **T [°C]** | **T [K]** |  | **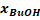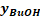** | **P° (bar)** | **γ** |
| --- | --- | --- | --- | --- | --- |
| **147** | 420 | 0 | 0 | 1.499 |  |
| **140.52** | 413.52 | 0.024 | 0.268 | 1.378 | 2.430 |
| **137.7** | 410.7 | 0.047 | 0.346 | 1.328 | 1.664 |
| **120.27** | 393.27 | 0.139 | 0.673 | 1.035 | 1.403 |
| **108.24** | 381.24 | 0.258 | 0.857 | 0.856 | 1.164 |
| **104.09** | 377.09 | 0.338 | 0.886 | 0.799 | 0.984 |
| **101.55** | 374.55 | 0.395 | 0.911 | 0.765 | 0.905 |
| **99.33** | 372.33 | 0.47 | 0.936 | 0.736 | 0.812 |
| **97.27** | 370.27 | 0.501 | 0.941 | 0.709 | 0.794 |
| **95.44** | 368.44 | 0.54 | 0.942 | 0.687 | 0.762 |
| **95.16** | 368.16 | 0.569 | 0.947 | 0.683 | 0.731 |
| **90.65** | 363.65 | 0.648 | 0.961 | 0.628 | 0.708 |
| **91.72** | 364.72 | 0.7046 | 0.966 | 0.641 | 0.641 |
| **91.07** | 364.07 | 0.73 | 0.967 | 0.633 | 0.627 |
| **86.69** | 359.69 | 1 | 1 | 0.583 | 0.515 |

**Table S4. Thermodynamic data**

1. Binary interaction parameters determined for the BuOH - BG binary using the NRTL model at different pressures.

| **Component i** | **BuOH** | **BuOH** | **BuOH** |
| --- | --- | --- | --- |
| **Component j** | BG | BG | BG |
| **Pressure [mbar]** | 300.00 | 700.00 | 1013.25 |
| **a_ij_** | -17.247 | -17.246 | -18.359 |
| **a_ji_** | -1.311 | 3.140 | 8.563 |
| **b_ij_ [K^-1^]** | 1547.6 | 7819.3 | 8273.0 |
| **b_ji_ [K^-1^]** | 470.8 | -1596.1 | -3848.5 |
| **α_ij_** | 0.3 | 0.3 | 0.3 |

1. Binary interaction parameters determined for the binary BG –W at atmospheric pressure, using NRTL (-) and UNIQUAC (--) models

|  | **NRTL** | **UNIQUAC** |
| --- | --- | --- |
| **Component i** | **W** | **W** |
| **Component j** | **BG** | **BG** |
| **a_ij_** | 1.792 | 0.852 |
| **a_ji_** | -2.643 | -1.105 |
| **b_ij_ [K^-1^]** | 563.40 | -204.19 |
| **b_ji_ [K^-1^]** | 1013.13 | -310.07 |
| **α_ij_** | 0.2 | --- |

(c) Thermal properties of glycolic acid

| Property | | Value | Reference |
| --- | --- | --- | --- |
| $\boldsymbol{T}_{\boldsymbol{fus}}$ [°C] | | 78.15 | (Emel’yanenko et al., 2010) |
| $\boldsymbol{\Delta}\boldsymbol{H}_{\boldsymbol{fus}}$ [J/mol] | | 19300 |  |
| $\boldsymbol{C}_{\boldsymbol{pL}}$ [J /mol K] | $A_{L}$ | 302.01 | Aspen plus V10 |
| $\boldsymbol{C}_{\boldsymbol{pL}}\boldsymbol{=}\boldsymbol{A}_{\boldsymbol{L}}\boldsymbol{+}\boldsymbol{B}_{\boldsymbol{L}}\boldsymbol{T}$ | $B_{L}$ | -0.2122 |  |
| [90 to 100 °C] | |  |  |
| $\boldsymbol{C}_{\boldsymbol{pS}}$ [J /mol K] | $A_{S}$ | 26.615 |  |
| $\boldsymbol{C}_{\boldsymbol{pS}}\boldsymbol{=}\boldsymbol{A}_{\boldsymbol{S}}\boldsymbol{+}\boldsymbol{B}_{\boldsymbol{S}}\boldsymbol{T}$ | $B_{S}$ | 0.3251 |  |
| [30 to 50 °C] | |  |  |

(d). Binary parameters for the NRTL model, obtained for the GA-W, GA-BuOH, and GA-BG systems.

| NRTL | | | |
| --- | --- | --- | --- |
| Component i | **GA** | **GA** | **GA** |
| Component j | **W** | **BG** | **BuOH** |
| a_ij_ | 0 | 0 | 0 |
| a_ji_ | 0 | 0 | 0 |
| b_ij_ [K^-1^] | 536.84 | -181.32 | 57.34 |
| b_ji_ [K^-1^] | -298.01 | 1317.20 | 554.90 |
| α_ij_ | 0.3 | 0.3 | 0.3 |

**Table S5: Optimized parameters with 95 % confidence for esterification of GA with butanol and Amberlyst 36 and Nafion NR50 as catalyst**

|  | Kinetic model For Amberlyst 36 | | | | | |
| --- | --- | --- | --- | --- | --- | --- |
| Parameter | PH | | LH | | ER | |
| k^+^ (mol.min^-1^.g_cata_^-1^) | 2.94.10^6^ |  | 5.32.10^10^ |  | 5.36.10^8^ |  |
| E_a_ (J.mol^-1^) | 53.04 |  | 56.81 |  | 56.00 |  |
| K_GA_ | - |  | - |  | - |  |
| K_BuOH_ | - |  | 1.10^2^ |  | 1.10^2^ |  |
| K_H20_ | - |  | 30.8 |  | - |  |
| K_BG_ | - |  | - |  | - |  |
| K_H2O_ | - |  | - |  | - |  |
| SRS | 0.99 |  | 0.52 |  | 0.62 |  |
| Err_rel_ (%) | 5.07 |  | 3.66 |  | 4.02 |  |

|  | Kinetic model for Nafion NR 50 | | | | | |
| --- | --- | --- | --- | --- | --- | --- |
| Parameter | PH | | ER | | LH | |
| k^+^ (mol.min^-1^.g_cata_^-1^) | 4.81.10^4^ | $\pm$ 2 | 1.10^6^ | $\pm$ 6.10^6^ | 3.10^6^ | $\pm$ 5.10^6^ |
| E_a_ (J.mol^-1^) | 48 | $\pm$ 2 | 47 | $\pm$ 2 | 47 | $\pm$ 2 |
| K_GA_ | - |  | 27 |  | 6 |  |
| K_BuOH_ | - |  | - |  |  |  |
| K_BG_ | - |  |  |  |  |  |
| K_H2O_ | - |  | - |  |  |  |
| SRS | 0.89 |  | 1.56 |  | 2.85 |  |
| Err_rel_ (%) | 6.59 |  | 5.42 |  | 9.01 |  |

**Table S6: Weisz modulus for batch experiments with Amberlyst 36, molar ratio butanol/GA of 1 :10**

| particule size (µm) | Weisz Modulus |
| --- | --- |
| 250 - 500 | 0.06 |
| > 500 | 0.08 |

**Table S7: experimental and simulated conversion, purity and recovery rate for feed flow of 0.6 kg.h^-1^, molar ratio butanol/GA : 1/10, R = 1 and 18 g of Nafion NR50 as catalyst**

|  | **Simulation** | **Experimental data** |
| --- | --- | --- |
| **GA conversion (%)** | **27** | **29** |
| **BG purity in boiler (%)** | **4.90** | **5.72** |
| **BG recovery rate (%)** | **99.23** | **100** |

**Figure S1: Stirring effect on glycolic acid conversion**


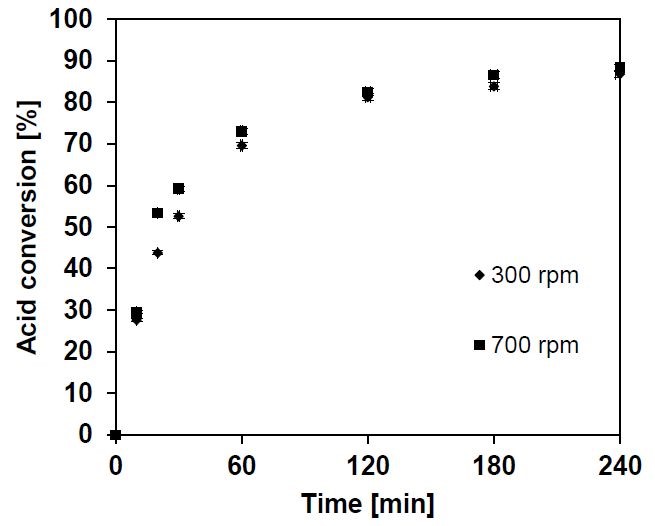


**Figure S2: Residue curve map of reactive mixture: glycolic acid, butanol, butyl glycolate and water at 150 mbar (a), 380 mbar (b) and 700 mbar (c). Distillation boundary (), feed composed at 40/60 butanol/glycolic acid molar ratio ()**


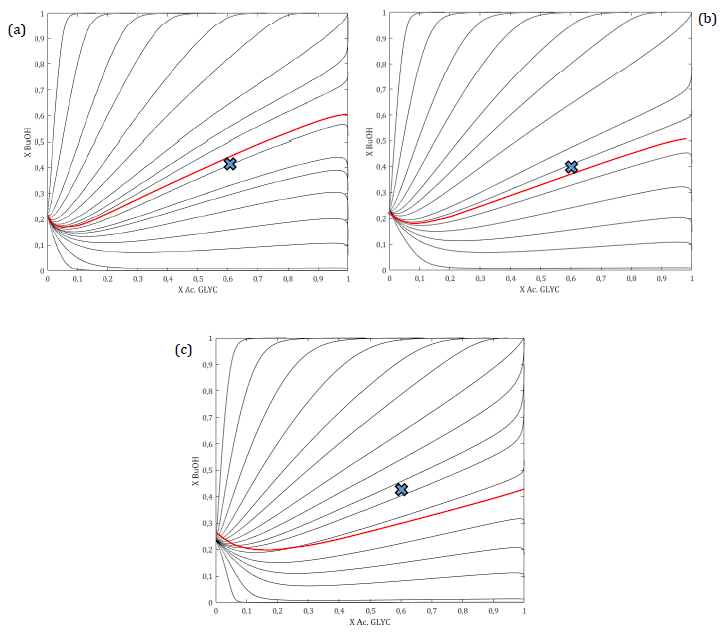


Figure S3: conversion, recovery rate and purity of ester in boiler vs feed stage.


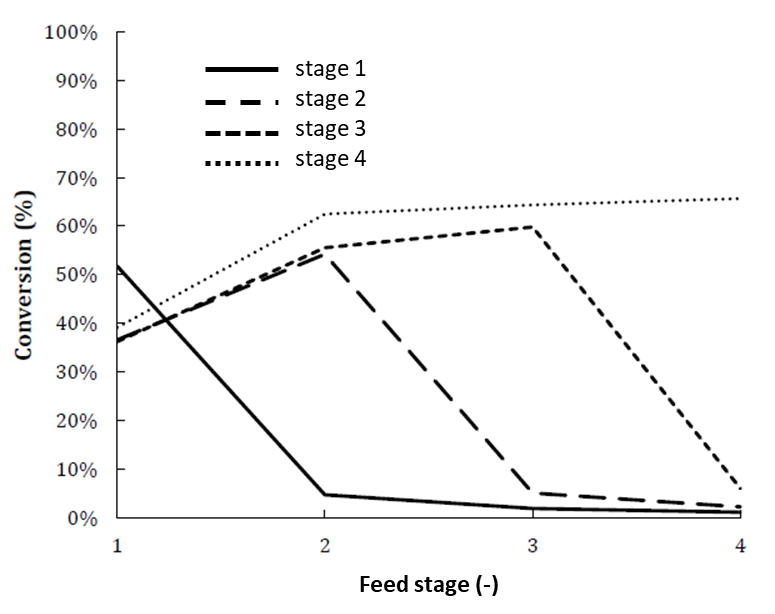


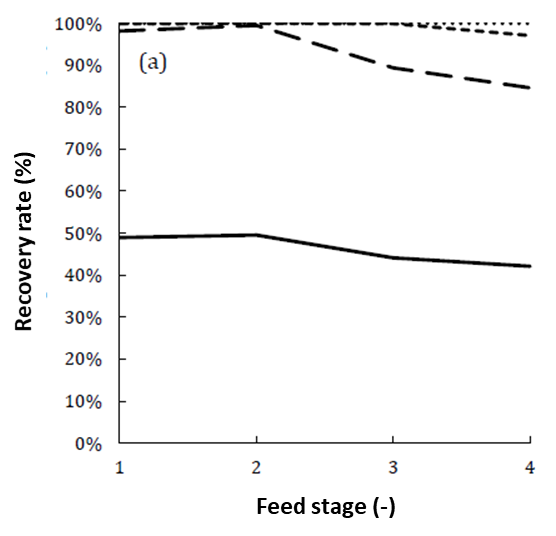

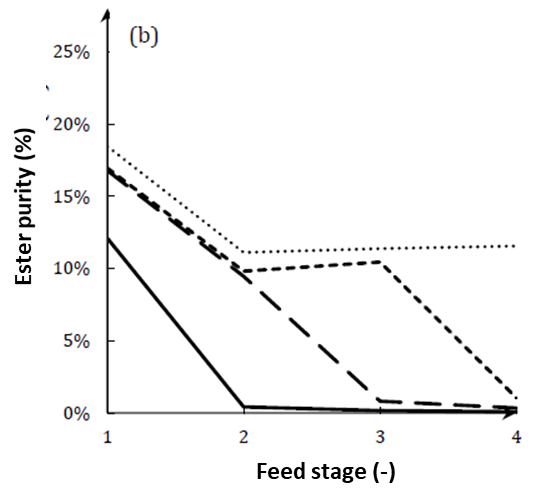

Supplement: Supplementary file 1 [file Table1.DOCX]
